# Epidemiology of postinjury multiple organ failure: a prospective multicenter observational study

**DOI:** 10.1007/s00068-024-02630-8

**Published:** 2024-09-12

**Authors:** Ryan S. Ting, Natasha A. Weaver, Kate L. King, Teagan L. Way, Pooria Sarrami, Lovana Daniel, Michael Dinh, Priya Nair, Jeremy Hsu, Scott K. D’Amours, Zsolt J. Balogh

**Affiliations:** 1 St George & Sutherland Clinical School, University of New South Wales, Sydney, NSW Australia; 2 University of Newcastle, Hunter Medical Research Institute, Newcastle, NSW Australia; 3 John Hunter Hospital, University of Newcastle, Hunter Medical Research Institute, Newcastle, NSW Australia; 4 NSW Institute of Trauma and Injury Management, NSW Agency for Clinical Innovation and University of New South Wales, South West Sydney Clinical School, Sydney, NSW Australia; 5 Westmead Hospital, University of New South Wales, South West Sydney Clinical School, Sydney, NSW Australia; 6 St Vincent’s Hospital, Sydney, Australia; 7 Westmead Hospital, Sydney, NSW Australia; 8 Liverpool Hospital Trauma and Acute Care Surgery Unit, University of New South Wales, South West Sydney Clinical School, Sydney, NSW Australia; 9 Department of Traumatology, Division of Surgery, John Hunter Hospital, Hunter Region Mail Centre, University of Newcastle, Locked Bag 1, Newcastle, NSW 2310 Australia

**Keywords:** Multiple organ failure, Polytrauma, Trauma, Trauma Center, Epidemiology

## Abstract

**Purpose:**

Postinjury multiple organ failure (MOF) is the sequela to the disease of polytrauma. We aimed to describe the contemporary population-based epidemiology of MOF within a mature trauma system, to analyse the time taken for MOF to develop, and to evaluate the temporal patterns and contributions of the individual constituent organ failures.

**Methods:**

Prospective observational study conducted across five Level-1 trauma centers in New South Wales, Australia. Trauma patients at-risk of MOF (Denver > 3 from 48 h post-admission), aged > 16 years, ISS > 15, and who stayed in ICU for ≥ 48 h were eligible for inclusion.

**Results:**

From May 2018–February 2021, 600 at-risk polytrauma patients were prospectively enrolled (mean(SD)age = 49(21)years, males = 453/600(76%),median(IQR)ISS = 26(20,34)). MOF incidence was 136/600(23%) among at-risk patients, 142/6248(2%) among major trauma patients (ISS > 12 per Australian definition), and 0.8/100,000 in the general population. The mortality rate was 55/600(11%) in the overall study population, and 34/136(25%) in MOF patients. 82/136(60%) of MOF patients developed MOF on day-3. No patients developed MOF after day-13. Among MOF patients, 60/136(44%) had cardiac failures (mortality = 37%), 39/136(29%) had respiratory failures (mortality = 23%), 24/136(18%) had renal failures (mortality = 63%), and 12/136(9%) had hepatic failures (mortality = 50%).

**Conclusion:**

Although a rare syndrome in the general population, MOF occurred in 23% of the most severely injured polytrauma patients. When compared to previous risk-matched cohorts, MOF become more common, but not more lethal, despite a decade older cohort. The heart has superseded the lungs as the most common organ to fail. Cardiac and respiratory failures occurred earlier and were associated with lower mortality than renal and hepatic failures.

**Supplementary Information:**

The online version contains supplementary material available at 10.1007/s00068-024-02630-8.

## Introduction

Postinjury multiple organ failure (MOF) is a syndrome that emerged 50 yearsago due to advancements in critical care that prevented trauma mortality from single organ failure, allowing the systemic inflammatory response, inter-organ cross-talk and septic complications to sequentially compromise further organ systems [1, 2]. 

Several clinical and epidemiological features of MOF went through documented change. MOF patients are older, less likely to sustain severe organ failure, and have a better chance of survival when compared to historical cohorts [3, 4]. Despite advances in trauma care, there is no consistent evidence of decreased incidence or less resource utilisation in MOF [4–6]. Both observational and interventional studies confirmed that MOF is the leading cause of late postinjury deaths, excessive intensive care unit length of stay and poor long-term functional outcomes for trauma patients [3, 4, 7]. 

Without specific treatment for MOF, key strategies are the application of preventative measures on the population at-risk and the provision of supportive therapy for dysfunctional and failing organs. For both strategies, it is essential to identify the predictors of MOF, especially the potentially modifiable predictors. This enables focused preventive strategies, which to date are mainly shock resuscitation related, to the right cohort [6, 8]. It also facilitates the power analysis and the targeted recruitment for potentially ground-breaking clinical trials on supportive therapies of MOF.

Most of our knowledge on MOF epidemiology, predictors and outcomes is based on either single institutional long-term prospective databases or large national registry-based studies. Institutional prospective databases provide valuable insight to long-term changes, but with any short-term timeframe, the sample size is low and the entire database’s results are flawed by any change of practice over a long-term, frequently over a decade, enrolment phase. Large national trauma registry studies can report on a greater number of subjects in shorter timeframes and are less likely to be affected by change in practice, but these works are retrospective, and the registries are not purpose-built for MOF research.

To address the need for a contemporary description of the population-based epidemiology of postinjury MOF, we performed a population-based, short inclusion term, prospective, multicenter observational study. We hypothesized that the incidence of postinjury MOF is lower than previously reported, with improved survival, and a change in the pattern of organ failure. We aimed to describe the contemporary incidence, onset, duration, and outcomes of MOF, including the contribution of individual organ failures to the syndrome, in the at-risk polytrauma patient population.

## Methods

### Study design and population

This was a prospective multicenter observational study performed at five Level-1 trauma centers (John Hunter Hospital, Liverpool Hospital, Royal Prince Alfred Hospital, St Vincent’s Hospital Sydney, and Westmead Hospital) in the state of New South Wales (NSW), Australia from May 2018 to February 2021. All eligible trauma patients were consecutively enrolled. To address our aim of reporting the contemporary population-based incidence of MOF, funding was sought to recruit the largest possible sample size in the shortest possible time, which resulted in the predetermined total sample size of 600 consecutive trauma patients.

All trauma patients in NSW at-risk of MOF are eventually admitted to one of the state’s seven Level-1 trauma centers. The state-wide incidence of MOF among major trauma patients (ISS > 12, Australian definition) was calculated from MOF incidence among the five participating Level-1 trauma centers against the known volume of severely injured patients admitted to all Level-1 trauma centers across NSW. This calculation was performed using the number of ISS > 12 patients as the denominator because our trauma registry defines major trauma as a binary variable, and is unable to report the number of ISS > 15 patients. Our inclusion criteria remains ISS > 15 to facilitate global comparisons.

The annual number of MOF cases was subsequently calculated, and the incidence of MOF per 100,000 people was extrapolated based on the population of NSW. MOF was defined as a Denver score > 3. The Denver score is the sum of grades allocated to pulmonary, renal, hepatic, and cardiac failures based on PaO2/FiO2 ratio, creatinine, total bilirubin, and inotrope use respectively [9]. The Denver > 3 after 48 h definition was used as it is a specific method of defining MOF that is well-validated in trauma cohorts, and is the same definition used in large purpose-built MOF databases, particularly in the USA and Australia, which will allow us to benchmark our progress in the prevention and management of MOF amidst 50 different definitions of MOF that has historically impeded this effort [5, 10]. 

MOF was not defined before 48 h to differentiate newly developing organ failure from the physiological derangements caused by the impact of injury and still incomplete resuscitation before 48 h. Ethical approval was granted by the Hunter New England Human Research Ethics Committee (Ref No 17/04/19/5.01 NSW HREC Ref No. LNR/17/HNE/100).

### Inclusion & exclusion criteria

Trauma patients aged ≥ 16 years with ISS > 15, who were admitted to the ICU, and stayed ≥ 48 h in ICU were eligible. Patients who sustained non-mechanical injury mechanisms (i.e. hanging/drowning/electrocution), or who had severe isolated neurological injuries were excluded.

### Data collected

At each site, patient and injury characteristics, shock parameters, hematological parameters, and blood gases on admission, worst values from 0 to 12 h and from 12 to 24 h post-admission, and outcomes (daily Denver score, daily Sequential Organ Failure Assessment (SOFA), ICU length of stay (LOS), ventilator days, mortality, and hospital LOS) were collected.

### Statistical analysis

Continuous variables were summarised by mean (SD) if the data distribution was normal, or median (IQR) if the data distribution was non-normal. Categorical variables were summarised by frequency count with percentage. Comparisons were performed using ANOVA F-tests for normal continuous variables, Kruskal-Wallis tests for non-normal continuous variables, or Chi-squared tests for categorical variables.

Early MOF was defined as a daily Denver > 3 that occurred on day 3 in ICU. Late MOF was defined as a daily Denver > 3 that was not present on day 3, but developed after an organ failure-free period following completed resuscitation [11]. The denominator for late MOF only included patients still in ICU on day 4.

Kaplan-Meier plots were generated to demonstrate time-to-MOF, which was the duration from admission to Day 1 of Denver > 3, and to provide estimates for the time taken until each individual organ failure (organ dysfunction grade of 3). Plots were generated to illustrate the outcomes of MOF patients with each constituent organ failure, showing time to organ failure, duration of failure, and whether this ended in discharge/death/censorship for each MOF patient. Patients were censored if they were still alive in ICU at 28 days with no MOF on any day prior. Analysis was performed using Stata version 18 (StataCorp, College Station, TX, USA).

## Results

The predetermined sample size of 600 was reached by consecutive recruitment from the five Level-1 trauma centers over 33 months (May 2018 to February 2021). In this timeframe, 6248 consecutive patients with major trauma were admitted to the five participating Level-1 trauma centers. The incidence of MOF among major trauma patients from recruited sites was 136/6248 (2%). From this figure, it was extrapolated that there were a total 182 cases of MOF across NSW based on the volume of major trauma admissions from participating and non-participating centers during the enrolment period. The annual incidence of MOF among the general population in NSW was calculated to be 60/8,000,000 people, or 0.8/100,000 people.

In this study population, the incidence of MOF among polytrauma patients determined to be at-risk was 136/600 (23%), and ranged from 16 to 27% between centers. Among MOF patients, the median (IQR) first day of MOF was Day 3 (3,5) post-admission. MOF lasted for a median (IQR) of 2 (1,4) days. The mean (SD) age among the overall population was 49 (21) years, and 453/600 (76%) patients were males. The median (IQR) ISS was 26 (20,34), 307/600 (51%) of included patients had an ISS > 25. Included patients stayed a median (IQR) of 7 (4,12) days in the ICU, and 20 (12,36) days in hospital. The overall mortality rate was 55/600 (11%), and ranged from 6% to 17% between centers (Table 1). MOF patients stayed a median (IQR) of 13 (9,21) days in the ICU, 27 (15,50) days in hospital, and had a mortality rate of 34/136 (25%).

**Table 1:** Characteristics and outcomes of polytrauma patients at-risk of MOF by site

Variable	Statistic	Center 1 (*N* = 180)	Center 2 (*N* = 186)	Center 3 (*N* = 83)	Center 4 (*N* = 19)	Center 5 (*N* = 132)	*P*-value	Total (*n* = 600)
Number of ISS > 12 admissions during enrolment period (Data from NSW Trauma Registry)	N	1580	1605	922	587	1554	-	6248
Age, years	mean (SD)	51 (21)	48 (21)	50 (22)	50 (21)	47 (19)	0.624	49 (21)
Sex, male	n (%)	129 (72%)	141 (76%)	64 (77%)	11 (58%)	108 (82%)	0.120	453 (76%)
ISS	median (IQR)	29 (22, 38)	24 (19, 29)	25 (19, 34)	26 (19, 29)	27 (22, 33)	0.002	26 (20, 34)
ISS > 25	n (%)	103 (57%)	79 (42%)	41 (49%)	10 (53%)	74 (56%)	0.047	307 (51%)
Head injury	n (%)	72 (40%)	77 (41%)	27 (33%)	4 (21%)	58 (44%)	0.217	238 (40%)
Systolic blood pressure on admission, mm Hg	mean (SD)	112 (28)	123 (29)	120 (37)	120 (32)	118 (32)	0.036	118 (31)
Had surgical procedure(s)	n (%)	148 (82%)	113 (61%)	56 (67%)	14 (74%)	118 (89%)	< 0.001	449 (75%)
Anaesthetic time, minutes	median (IQR)	300 (120, 573)	150 (0, 345)	155 (0, 285)	0 (0, 195)	367 (157, 647)	< 0.001	210 (0, 457)
Comfort-focused care	n (%)	9 (5.0%)	9 (4.8%)	11 (13%)	1 (5.3%)	12 (9.1%)	0.078	42 (7.0%)
Incidence of MOF (Denver > 3 after 48 h)	n (%)	48 (27%)	37 (20%)	17 (20%)	3 (16%)	31 (24%)	0.517	136 (23%)
Early MOF incidence (Denver > 3 on day 3)	n (%)	31 (17%)	23 (12%)	8 (9.6%)	2 (11%)	16 (12%)	0.436	80 (13%)
Late MOF incidence (first Denver > 3 days 4–10)^a^	n (%)	14/108 (13%)	9/139 (6.5%)	7/67 (10%)	1/10 (10%)	13/74 (18%)	0.159	44/398 (11%)
Maximum Denver Score	median (IQR)	3 (2, 4)	2 (0, 4)	2 (0, 4)	2 (0, 3)	3 (2, 4)	< 0.001	2 (1, 4)
Maximum SOFA Score	median (IQR)	8 (6, 11)	5 (2, 10)	7 (4, 9)	8 (3, 11)	8 (6, 10)	< 0.001	7 (4, 10)
Ventilator days	median (IQR)	4 (2, 9)	2 (0, 5)	2 (0, 5)	3 (0, 9)	5 (3, 11)	< 0.001	3 (0, 8)
Ventilator free days	median (IQR)	16 (10, 30)	15 (29, 29)	12 (7, 23)	22 (8, 37)	18 (9, 34)	0.056	15 (9, 29)
ICU LOS	median (IQR)	8 (5, 13)	6 (4, 10)	5 (4, 7)	5 (3, 13)	9 (6, 17)	< 0.001	7 (4, 12)
Hospital LOS	median (IQR)	23 (14, 39)	17 (11, 34)	13 (9, 28)	25 (8, 50)	25 (14, 41)	< 0.001	20 (12, 36)
Mortality	n (%)	11 (6.1%)	14 (7.5%)	14 (17%)	1 (5.3%)	15 (11.%)	0.047	55 (11%)
	**Mechanism of Injury**							
Motor vehicle collision	n (%)	88 (49%)	55 (30%)	8 (9.6%)	4 (21%)	41 (31%)	-	196 (33%)
Fall/jumped	n (%)	24 (13%)	57 (31%)	29 (35%)	6 (32%)	22 (17%)	-	138 (23%)
Motorbike collision	n (%)	35 (19%)	28 (15%)	6 (7.2%)	2 (11%)	22 (17%)	-	93 (16%)
Pedestrian	n (%)	15 (8.3%)	15 (8.1%)	11 (13%)	2 (11%)	23 (17%)	-	66 (11%)
Assault/penetrating	n (%)	7 (3.9%)	10 (5.4%)	16 (19%)	3 (16%)	10 (7.6%)	-	46 (7.7%)
Cyclist	n (%)	4 (2.2%)	6 (3.2%)	7 (8.4%)	2 (11%)	4 (3.0%)	-	23 (3.8%)
Deliberate self-harm	n (%)	7 (3.9%)	3 (1.6%)	4 (4.8%)	2 (11%)	5 (3.8%)	-	21 (3.5%)
Crush	n (%)	4 (2.2%)	4 (2.2%)	1 (1.2%)	0	5 (3.8%)	-	14 (2.3%)
Other	n (%)	3 (1.7%)	11 (5.9%)	5 (6.0%)	0	5 (3.8%)	-	24 (4.0%)

^a,^ The denominator for late MOF only includes patients still in ICU on day 4

### Analysis of time to MOF

**Fig. 1:**
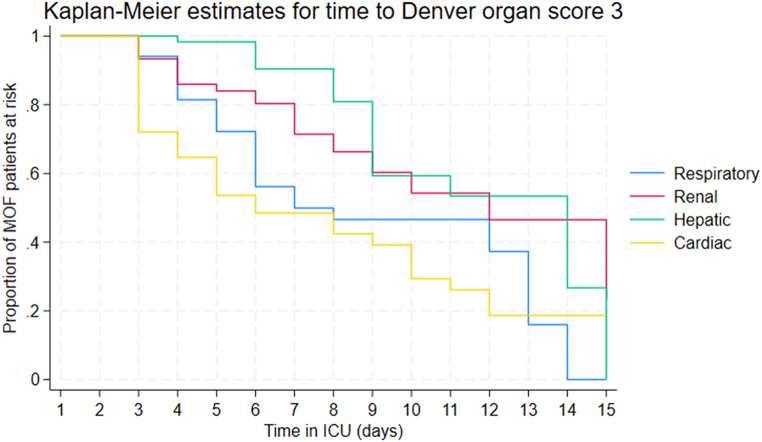
Kaplan-Meier plot showing time to each organ failure (Grade 3 dysfunction > 48 h) among patients who developed MOF (Denver score > 3 after 48 h) over the first 15 days where MOF developed

**Fig. 2:**
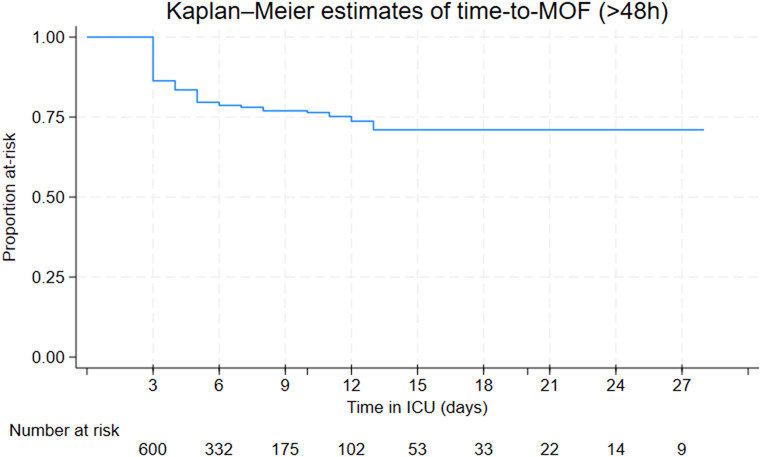
Kaplan-Meier plot showing proportion of patients at-risk of MOF over time (after 48 h) in ICU

**Fig. 3:**
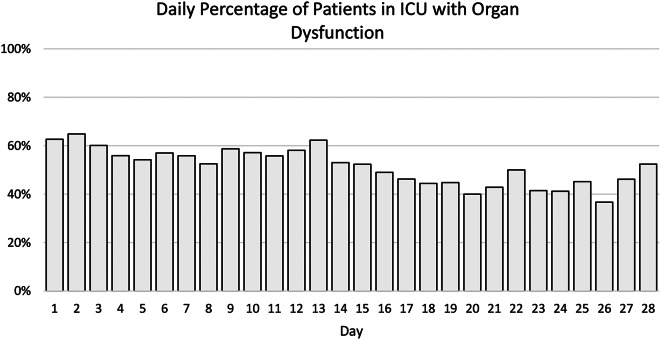
Percentage of included patients who remained in the ICU each day with organ dysfunction

**Fig. 4:**
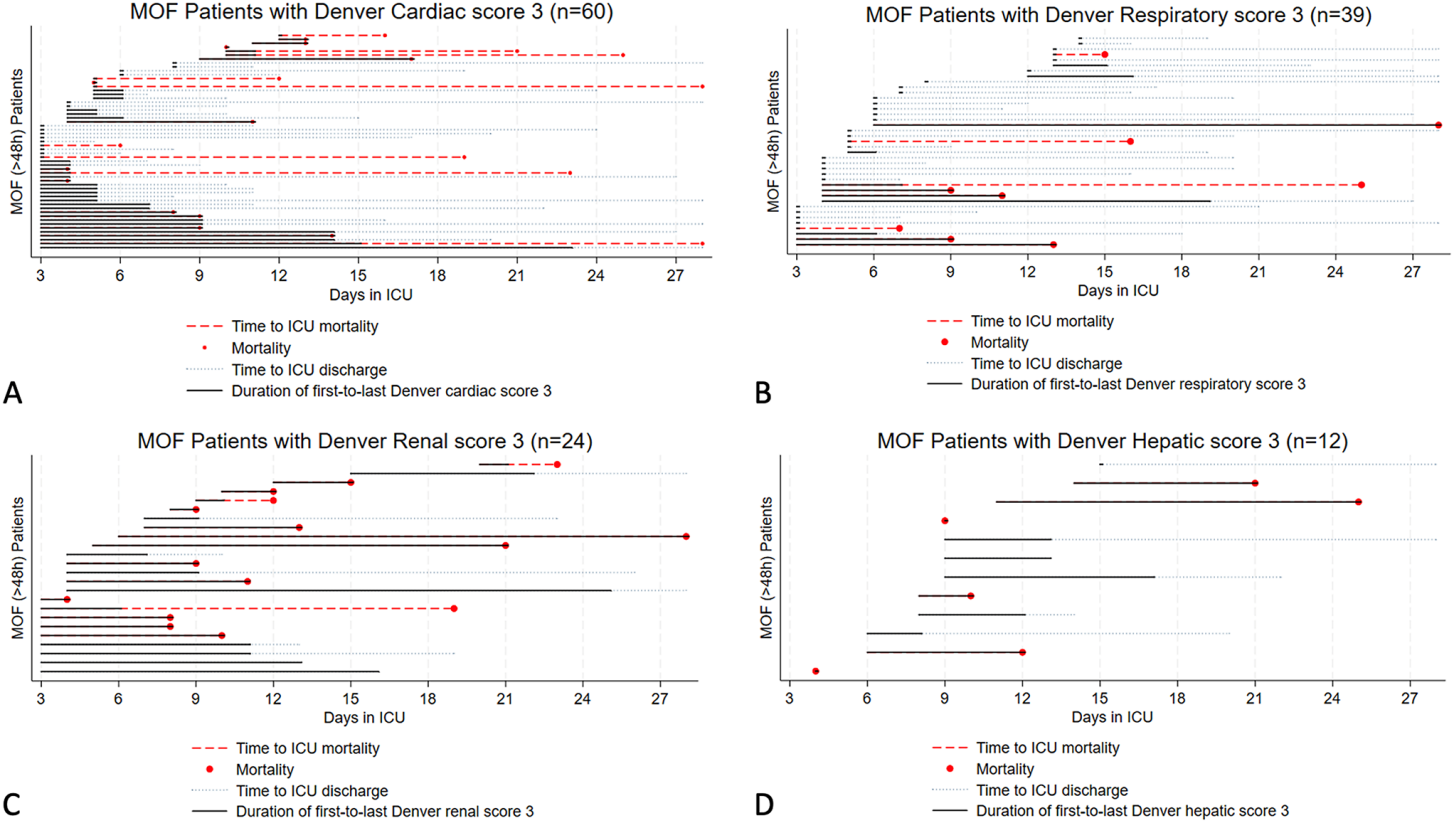
Individual MOF (> 48 h) patient timelines to (**A**) cardiac, (**B**) respiratory, (**C**) renal, and/or (**D**) hepatic failure (Grade 3 dysfunction > 48 h respectively), ICU discharge or mortality, sorted by time to organ failure, and duration of organ failure. Of the 136 patients who developed MOF (Denver > 3 after 48 h), 93/136 (68%) developed grade 3 dysfunction in at least one of the included organs after 48 h. The most common organs affected were the heart (*n* = 60), and the lungs (*n* = 39), followed by the kidneys (*n* = 24) and liver (*n* = 12) (Fig. 5)

Kaplan-Meier estimates for time to first grade 3 dysfunction (> 48 h) for each individual organ among the patients who developed MOF showed that cardiac and respiratory failures occurred earliest, from day 3. Cardiac failures occurred earlier than respiratory failures, and were closely followed by renal failures. Hepatic failures were a late manifestation of MOF (Fig. 1). Early MOF was present in 82/136 (60%) of all MOF patients (Supplement File 1, Supplement File 2). No patients developed MOF after day 13 (Fig. 2). There was no apparent second peak in MOF onset beyond the initial days after admission. On average, 57% of the at-risk patients who remained in the ICU had some form of organ dysfunction (Denver > 0) each day (Figs. 3 and  4)

**Fig. 5:**
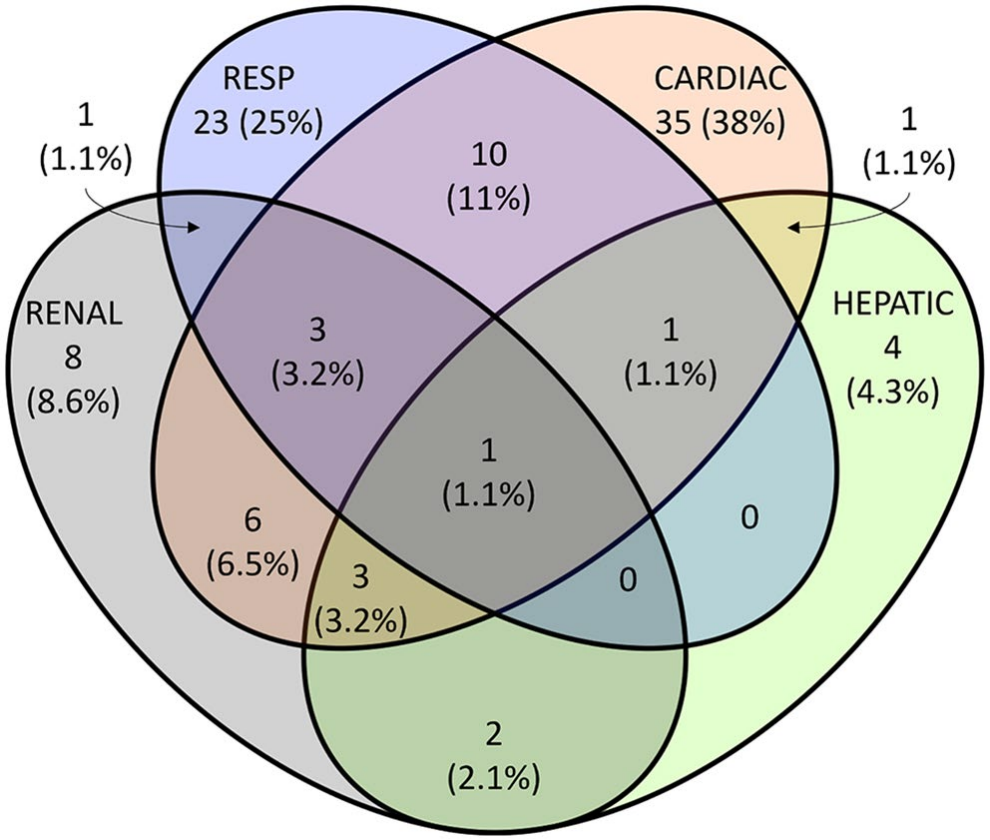
Multiple Venn diagram showing the counts (%) and combinations of organ failure (Grade 3 dysfunction). Percentages expressed with total number of patients with Grade 3 dysfunction after 48 h (*n* = 93) as the denominator

Cardiac and respiratory failures were the most prevalent organ failures among those who developed MOF (Fig. 5). However, mortality was lower among MOF patients with cardiac (37%) and respiratory (23%) failures than patients with renal (63%) and hepatic failures (50%). Patients with respiratory failures had the longest ICU stays at median (IQR) 19 (11,27) days, and the longest hospital stays at median 28 (16,57) days (Supplement File 3).

## Discussion

MOF is a high mortality rare syndrome by any definition. The annual incidence of MOF was 0.8/100,000 people in NSW, Australia’s most densely populated state, 2% among all major trauma patients, or 23% among the most severely injured at-risk polytrauma patients. Compared to previous cohorts that used the same MOF definition (Denver > 3 after 48 h) and study inclusion criteria, the contemporary incidence of MOF has increased, and in a now a decade older patient population alongside the documented shift from crystalloid to haemostatic resuscitation [6, 12, 13]. This is an extremely high-acuity syndrome. MOF patients stayed a median of 13 days in ICU, 27 days in hospital, with a mortality rate of 25%.

The definition of a rare disease varies between regions and organisations. Among heterogenous prevalence thresholds, it was found that the average definition threshold for a rare disease is 40–50/100,000 people [14]. This qualifies postinjury MOF as a truly rare syndrome among the general population. In contrast, a quarter of the most severely injured patients are expected to develop MOF during their ICU admission. Therefore, MOF demands specific focus for research and advocacy for funding as a sequela to the disease of polytrauma [15, 29].

This study demonstrated that as our population evolves, so too does the epidemiology of MOF. In this severely injured cohort (median ISS = 26), the incidence of MOF was 23%, and the overall mortality rate was 11%. In comparison to previous investigations that used the same MOF definition (Denver > 3) and study inclusion criteria, the incidence of MOF in this cohort was higher than the 13% and 14% incidences of MOF reported by the Denver group between 1990 and 1993 and 2003–2010 respectively [4, 16], and the 15% reported by the Newcastle group between 2007 and 2012 [3]. Expectedly, the overall incidence of MOF in our study was lower than the 33% reported by Frohlich in their analysis of 31,154 patients from the German trauma registry (TR-DGU) between 2002 and 2011 [17]. This may have been due to their use of the more sensitive SOFA to define MOF, and unlike the present study, they also included MOF that was diagnosed within the first 48 h [10]. Interestingly, a recent study by Cole et al. [18]. found that the incidence of MOF was 56% in a point-prevalence study conducted across 29 UK major trauma centers, although they used the same MOF definition of SOFA > 6 (including organ dysfunctions before 48 h, unlike most American and Australian studies) as Frohlich et al. [17]. 

The 11% overall mortality rate in this study was lower than in the cohort of 31,154 TR-DGU patients by Frohlich et al. [17]. (mortality = 16%, mean ISS = 28), similar to the rate reported by the Denver group (mortality = 8-11%, mean ISS = 24–33), and higher than the rate reported by the Newcastle group earlier (mortality = 6%, mean ISS = 30) [3, 4, 16, 19]. 

Males comprised 76% of our study population, and continue to predominate the polytrauma cohort. In keeping with temporal trends, the age of patients at-risk continues to rise, with our results suggesting that these polytrauma patients are a decade older than they would have been 20 years ago [17, 19, 20]. Indirectly, a similar mortality rate despite increasing age and its associated comorbidities suggests that our management of MOF is improving.

This study did not identify the apparent historically reported second peak in MOF onset beyond the first few days in hospital. Kaplan-Meier modelling of time-to-MOF demonstrated that with contemporary trauma care, at-risk polytrauma patients would be unlikely to develop postinjury MOF outside of the first fortnight. Seminal reports had initially described postinjury MOF as a bimodal phenomenon, where late MOF, which occurred after the patient was physiologically stabilized following the initial insult, then proceeded to develop MOF after day 3, was postulated to be triggered by ‘second hits’ [11, 21]. The historic second peak in MOF tended to be due to sepsis, primarily abdominal sepsis, and we did not see that in our population. Other potential contributors may include general improvements in critical care, less catheter, maxillofacial sinus, and acalculous cholecystitis related sepsis, and advances in wound management techniques such as freely draining open wounds under vacuum sealed dressings instead of soaked gauze dressings changed in the ICU. Ultimately, we do not know the exact reason why MOF has evolved a unimodal phenomenon, and we may not be able to answer this unless a center sees bimodal distribution.

Our findings corroborate more recent studies showing that the onset of MOF for the most part is early, with 59% of patients in this study developing MOF day 3 [3, 20, 22]. This suggests that our efforts should be focussed on preventing MOF early. Although MOF has evolved into a uniformly early phenomenon, we found that it also represents a continuous burden of low-grade organ dysfunction. The daily proportion of patients from this study who remained in the ICU with organ dysfunction (Denver > 0) was 57%. This residual sub-MOF organ dysfunction is common, and is a potential major contributor to resource consumption in these patients.

We found that the heart was the first and most common organ to fail, in contrast to historical precedents, where the lungs had been reported to be the first and most common organ to fail. Regel et al. [23]. found that the lungs were the first to fail in 20/39 (64%) MOF patients (74% MOF patients had lung failures overall), whereas the heart was the first organ to fail in just 5/39 (13%) of MOF patients (21% of MOF patients had cardiac failures overall). Faist et al. [24]. postulated that MOF was not possible without the catalyst of respiratory failure. In their study of 433 polytrauma patients, 8% developed MOF, all of whom developed respiratory failure, among whom the mortality was 55%. In contrast, cardiac failures occurred in only 12% of MOF patients, although mortality in this subgroup was 75%. Two recent short inclusion term prospective cohort studies conducted in the United Kingdom reported that respiratory and cardiac failures were the first and second most common organ failures in 245 MOF patients (97% respiratory and 91% cardiac) [25] and 860 MOF patients (92% respiratory and 77% cardiac) respectively per the SOFA definition [18]. By comparison, only 39/136 (29%) of our MOF patients acquired respiratory failure. However, patients with respiratory failure had the longest hospital stays. Patients with renal and hepatic failures had the highest mortality. This may be because cardiac and respiratory failures were earlier phenomena, meaning that by the time the kidneys and liver failed, that the odds of death were higher. Furthermore, the threshold to define kidneys and liver failure may be disproportionately higher relative to the cardiac and respiratory failure on the spectrum of dysfunction compatible with life [26–28]. While these possible explanations are speculative at this stage, this study has identified the epidemiology of each individual organ failure, which can serve to improve definitions and investigate specific causes of organ failures in isolation and in combinations.

This study had several limitations. Although differences in patient management can occur between centers, one aim of this observational study was to describe the contemporary population-based epidemiology of MOF to provide a benchmark our progress in the management of MOF, provide a baseline for future power analyses. Whilst the Denver > 3 is a sensitive, validated and frequently utilized MOF definition in trauma cohorts, it has lower sensitivity than the SOFA, which is another common definition more frequently used for mixed ICU cohorts. While all participating centres were oriented to record inotrope medication only when used for supporting cardiac contractility instead of systemic vascular resistance or cerebral perfusion pressure, there is a chance of slight over-reporting of cardiac failure. Statistical modelling of time-to-MOF as binary phenomenon are blunt measures of continuous biological processes that fluctuate around a threshold on any scoring system.

## Conclusion

MOF is a rare syndrome, affecting 0.8/100,000 in the general population annually, but 23% of severely injured polytrauma patients, from which 25% would not survive to hospital discharge. The epidemiology of MOF has evolved with an aging, more comorbid at-risk population, and the pattern of organ failure is now characterised by early cardiac failures, in contrast to historical reports where pulmonary failures were the catalyst for MOF. Cardiac and respiratory failures tended to occur earlier, and were associated with lower mortality than later occurring renal or hepatic failures. MOF continues to be a rare, but highly lethal and resource-intensive syndrome, for which we clearly identified the target population and incidence to optimise recruitment for future interventional studies, and to benchmark as a target for MOF prevention. Like any rare, lethal syndrome, MOF requires focused multi-institutional efforts supported by dedicated funding and researchers.

## Electronic supplementary material

Below is the link to the electronic supplementary material.

Supplementary Material 1

Supplementary Material 2

Supplementary Material 3

## Data Availability

No datasets were generated or analysed during the current study.
