# Supplementary material for: Epidemiology of postinjury multiple organ failure: a prospective multicenter observational study
Source: Eur J Trauma Emerg Surg. 2024 Sep 12;50(6):3223–31. doi: 10.1007/s00068-024-02630-8 (PMC11666632; doi:10.1007/s00068-024-02630-8)
Supplement: Supplementary file 1 — Supplementary Material 1 [file 68_2024_2630_MOESM1_ESM.pdf]

Article Title: Epidemiology of Postinjury Multiple Organ Failure: A Prospective Multicenter Observational Study

Journal Name: European Journal of Trauma and Emergency Surgery

Author Names: Ryan S. Ting, Natasha A. Weaver, Kate L. King, Teagan L. Way, Pooria Sarrami, Lovana Daniel, Michael Dinh, Priya Nair, Jeremy Hsu, Scott K. D'Amours, Zsolt J. Balogh

Corresponding Author: Zsolt J. Balogh

Affiliation: John Hunter Hospital and University of Newcastle, Hunter Medical Research Institute, Newcastle, New South Wales, Australia

Email: Zsolt.Balogh@health.nsw.gov.au

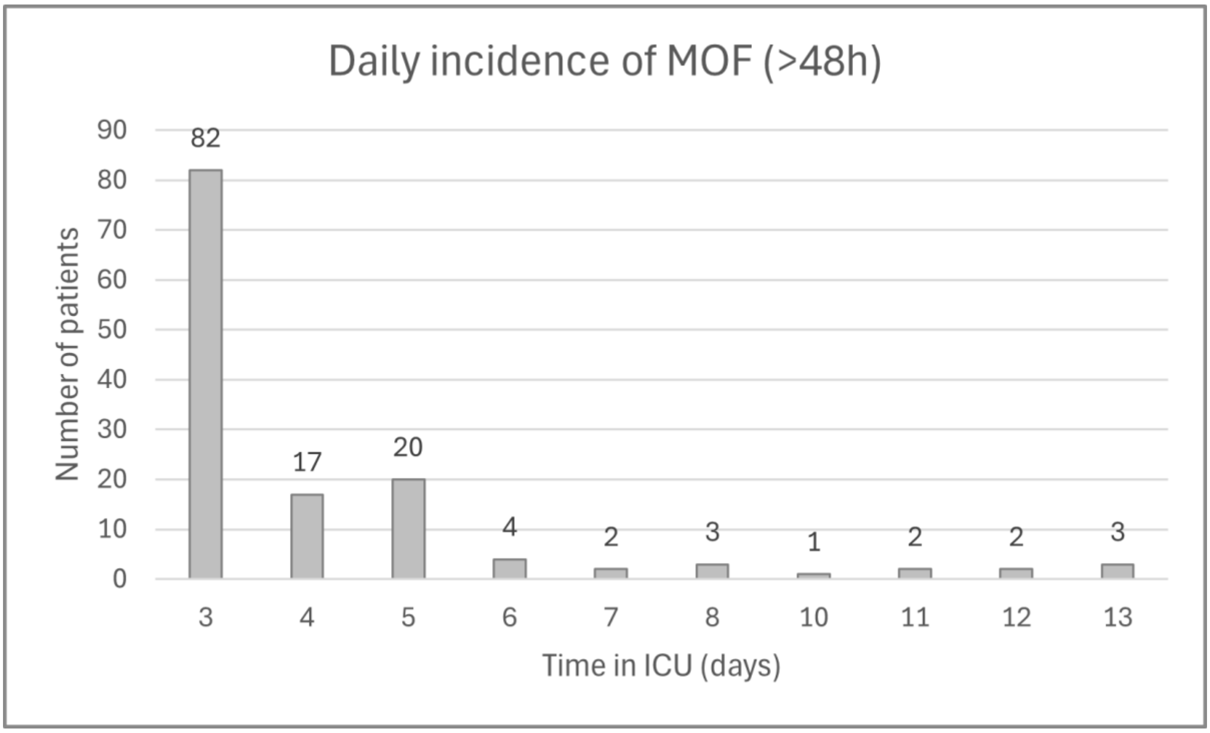

Supplement File 1. Daily incidence of MOF in the study population.
