# Supplementary material for: Epidemiology of postinjury multiple organ failure: a prospective multicenter observational study
Source: Eur J Trauma Emerg Surg. 2024 Sep 12;50(6):3223–31. doi: 10.1007/s00068-024-02630-8 (PMC11666632; doi:10.1007/s00068-024-02630-8)
Supplement: Supplementary file 3 — Supplementary Material 3 [file 68_2024_2630_MOESM3_ESM.pdf]

Article Title: Epidemiology of Postinjury Multiple Organ Failure: A Prospective Multicenter Observational Study

Journal Name: European Journal of Trauma and Emergency Surgery

Author Names: Ryan S. Ting, Natasha A. Weaver, Kate L. King, Teagan L. Way, Pooria Sarrami, Lovana Daniel, Michael Dinh, Priya Nair, Jeremy Hsu, Scott K. D'Amours, Zsolt J. Balogh

Corresponding Author: Zsolt J. Balogh

Affiliation: John Hunter Hospital and University of Newcastle, Hunter Medical Research Institute, Newcastle, New South Wales, Australia

Email: Zsolt.Balogh@health.nsw.gov.au

Supplement File 3. MOF patient outcomes for each individual organ failure (Grade 3 dysfunction after 48hours).

	**Number of patients with Grade 3 Dysfunction (>48h)**	**Mortality in ICU, n (%)**	**ICU LOS, median (IQR), days**	**Hospital LOS, median (IQR), days**
Cardiac	60	22 (37%)	13 (9, 23)	27 (15, 52)
Respiratory	39	9 (23%)	19 (11, 27)	28 (16, 57)
Renal	24	15 (63%)	13 (10, 22)	23 (13, 38)
Hepatic	12	6 (50%)	17 (11, 24)	23 (13, 62)
